# Sleep Duration and Sleep Quality in Caregivers of Patients With Dementia: A Systematic Review and Meta-analysis

**DOI:** 10.1001/jamanetworkopen.2019.9891

**Published:** 2019-08-23

**Authors:** Chenlu Gao, Nikita Y. Chapagain, Michael K. Scullin

**Affiliations:** 1 Department of Psychology and Neuroscience, Baylor University, Waco, Texas

## Abstract

**Importance:**

In the United States, 16 million family caregivers provide long-term care for patients with dementia. Although one’s physical, mental, and cognitive health depends on sleep, many caregivers experience chronic stress, and stress is typically associated with worse sleep quantity and quality.

**Objective:**

To quantify the extent, nature, and treatability of sleep problems in dementia caregivers.

**Data Sources:**

PubMed and Scopus databases were systematically searched for articles published through June 2018 using the following keywords: *caregiver* or *spouse* or *caretaker* AND *sleep* or *circadian* AND *dementia* or *Alzheimer*. Backward citation tracking was performed, and corresponding authors were contacted for additional data to conduct meta-analyses and pooled analyses.

**Study Selection:**

Two reviewers independently screened 805 studies to identify those that reported sleep duration or sleep quality in caregivers of patients with dementia.

**Data Extraction and Synthesis:**

Following the PRISMA guidelines, 2 reviewers independently extracted data from all studies and conducted National Heart, Lung, and Blood Institute study quality assessments. Meta-analyses with random-effects models were performed to evaluate sleep duration, sleep quality, and sleep interventions in dementia caregivers.

**Main Outcomes and Measures:**

Sleep quality and total sleep time were measured by polysomnography, actigraphy, and self-report.

**Results:**

Thirty-five studies were analyzed with data from 3268 caregivers (pooled mean age [SD of sample means], 63.48 [5.99] years; 76.7% female) were analyzed. Relative to age-matched control noncaregiver adults, caregivers had lower sleep durations akin to losing 2.42 to 3.50 hours each week (Hedges *g* = −0.29; 95% CI, −0.48 to −0.09; *P* = .01). Sleep quality was significantly lower in caregivers (Hedges *g* = −0.66; 95% CI, −0.89 to −0.42; *P* < .001), but caregivers who underwent sleep intervention trials had better sleep quality than caregivers who did not receive a sleep intervention (Hedges *g* = 0.35; 95% CI, 0.20-0.49; *P* < .001).

**Conclusions and Relevance:**

Sleep debt is known to have cumulative associations with physical, mental, and cognitive health; therefore, poor sleep quality in dementia caregivers should be recognized and addressed. Although the caregiving role is stressful and cognitively demanding by its nature, better sleep quality was observed in caregivers who received low-cost behavioral interventions.

## Key Points

**Question:**

Is caregiving for patients with dementia associated with shorter or poorer-quality sleep and treatable by behavioral interventions?

**Findings:**

Based on the systematic review and meta-analysis of 35 studies (3268 caregivers), caregivers lost 2.42 to 3.50 hours of sleep each week due to difficulty falling asleep and maintaining sleep, a significant difference relative to age-matched noncaregiver controls. However, significantly better sleep quality was observed in caregivers after behavioral interventions.

**Meaning:**

Many caregivers of patients with dementia have chronic sleep problems, but implementing behavioral sleep interventions is associated with better sleep quality in this population.

## Teaser

This systematic review and meta-analysis quantifies the extent, nature, and treatability of sleep problems in caregivers of patients with dementia.

## Introduction

Dementia affects approximately 50 million adults globally, with this number expected to increase by 2050 to 131 million.^1^ The global annual cost of dementia is approaching US $1 trillion,^2^ in large part due to patients’ loss of independent functioning, such as problems with eating, bathing, grooming, incontinence, and memory loss. In the United States, this lack of independent functioning has spurred 16 million adults to become informal caregivers to their spouse, parent, or friend who has Alzheimer disease or a related dementia.^3^ Caregiving for patients with dementia is akin to adding a part-time job. The Alzheimer’s Association estimates that family members on average spend 21.9 hours per week caring for those with dementia.^3^

Caregiving is emotionally and cognitively demanding, and an accumulating literature indicates that caregivers’ overall health is adversely altered.^4^ Cognitive functioning may also decline in caregivers, with studies reporting lower performance relative to age-matched control adults on tests of verbal memory^5^ and attention.^6^ One possibility is that sleep disturbances exacerbate the observed changes to mental, physical, and cognitive health.^4^ For example, mechanistic work has shown that acute and chronic sleep problems are associated with difficulties regulating stressors, quicker cortical thinning, and lower glymphatic clearance of β-amyloid from interstitial space.^7,8,9^

The objective of the present systematic review and meta-analysis was to unify and quantify the collective literature on sleep in dementia caregivers. We considered multiple theoretical views. First, the controversial sleep need view is that older adults need less sleep than young adults; by this view, caregivers should report less total sleep time but without changes in perceived sleep quality.^8^ Second, based on the caregiver empowerment model, which argues that caregiving is a positive, enriching experience (eg, due to social bonding),^10^ sleep quality should be unchanged or even improved in caregivers. Third, based on the environmental stressor view that caregiver stress is externally driven (ie, by the patient and/or situation), caregivers should show worse sleep quality that does not change even with caregiver-focused sleep interventions (ie, because the intervention does not remove the environmental stressor).^4^ Fourth, based on the stress and coping model’s view that caregiver health problems are driven by maladaptive responses to stressors (eg, increased alcohol, decreased exercise, and altered health behaviors), sleep interventions should be associated with better sleep quality in caregivers.^11^

## Methods

### Overview and Data Sources

**Figure 1.**
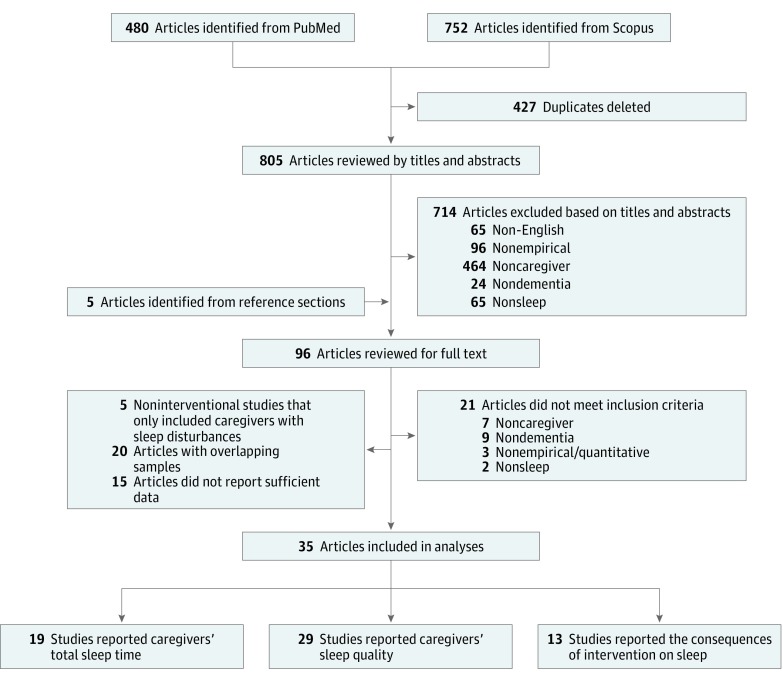
PRISMA Diagram of the Study Selection Process PRISMA indicates Preferred Reporting Items for Systematic Reviews and Meta-analyses.

Approval from the Baylor University Institutional Review Board was waived because this study used existing, anonymized data. We followed the Preferred Reporting Items for Systematic Reviews and Meta-analyses (PRISMA) reporting guideline.^12^ As a first step, we searched PubMed and Scopus databases for articles published through June 2018 using the following keywords: *caregiver* or *spouse* or *caretaker* AND *sleep* or *circadian* AND *dementia* or *Alzheimer*. Every retrieved article was further examined to identify relevant studies. The selection process is shown in Figure 1.

### Study Selection

In total, 805 articles were screened independently by 2 reviewers (C.G. and N.Y.C.) for meeting the inclusion criteria. If the 2 reviewers did not reach consensus, a third reviewer (M.K.S.) decided whether a study should be included. We used the following 5 criteria to determine study inclusion: (1) published in English, (2) published in a peer-reviewed journal, (3) recruited at least 1 group of caregivers of patients with dementia, (4) quantitatively assessed either sleep duration or sleep quality in caregivers, and (5) did not list sleep disturbance as an inclusion criterion for noninterventional studies.

When multiple articles referred to the same set of data, we selected the article with the most comprehensive data. If means and standard deviations for sleep duration or sleep quality were not reported, we contacted the authors to request the descriptive data. Nine of the 24 authors we contacted shared their data. Thirty-five articles were included in the analyses.

### Data Extraction

Two research personnel (C.G. and N.Y.C.) independently reviewed all studies that met the inclusion criteria and extracted relevant data. Disagreements were resolved by discussion among the reviewers. eTable 1 in the Supplement lists the extracted variables. For studies that implemented a sleep intervention for caregiver individuals, the baseline sleep data were also included in cross-sectional analyses.

### Quality Assessment

We assessed the quality of each study using standardized instruments from the National Heart, Lung, and Blood Institute (NHLBI).^13^ Depending on the study design, we used the NHLBI quality assessment tools for observational cohort and cross-sectional studies (eTable 2 in the Supplement), before-after studies with no control group (eTable 3 in the Supplement), or controlled intervention studies (eTable 4 in the Supplement). Two reviewers (C.G. and N.Y.C.) independently rated each quality domain for every study, and discrepancies were resolved by a third rater (M.K.S.).

### Sleep Measures

Total sleep time was measured by polysomnography (PSG), actigraphy, or self-report. Polysomnography measures sleep via electroencephalography, eye movements, and muscle tone. Actigraphy measures movement via wristworn accelerometers to estimate sleep/wake state. Sleep quality was typically assessed by the Pittsburgh Sleep Quality Index (PSQI), a self-report measure. The PSQI is a 9-item questionnaire that assesses subjective sleep quality for the past month.^14^ The PSQI global scores range from 0 to 21, with higher scores indicating poorer sleep quality. When data were available, the global scores were further separated into the following 7 component scores: subjective sleep quality, sleep latency, sleep duration, habitual sleep efficiency, sleep disturbances, use of sleep medication, and daytime dysfunction (each ranges in score from 0 to 3). Self-reported sleep quality was also sometimes assessed by similar questionnaires, including the Morin Daily Sleep Diary, the Pittsburgh Sleep Disorders Questionnaire, and the Sleep Problems Questionnaire (Table 1).

**Table 1.** Studies That Reported Total Sleep Time (19 Studies) and Sleep Quality (29 Studies) Separated by Caregiver and Control Data

Source	No.		Total Sleep Time Measure	Sleep Quality Measure	Included in Meta-analysis of Total Sleep Time	Included in Meta-analysis of Sleep Quality
	Caregivers	Controls				
Wilcox and King,^15^ 1999	90	NA	PSQI	PSQI	No	No
King et al,^16^ 2002	94	NA	PSQI	PSQI	No	No
Caswell et al,^17^ 2003	44	66	NA	SPQ	No	Yes
Ancoli-Israel et al,^18^ 2005	63	NA	NA	PSQI	No	No
McCurry et al,^19^ 2005	36	NA	Actigraphy	PSQI	No	No
McKibbin et al,^20^ 2005	73	40	PSG	PSQI	Yes	Yes
Vitaliano et al,^21^ 2005	96	95	NA	PSDQ	No	Yes
Brummett et al,^22^ 2006	175	169	NA	PSQI	No	Yes
Mausbach et al,^23^ 2006	40	NA	PSG	NA	No	No
Willette-Murphy et al,^24^ 2006	37	37	Sleep diary	MDSD	Yes	Yes
Adachi et al,^25^ 2007	42	NA	NA	PSQI	No	No
Lee et al,^26^ 2007	39	NA	Actigraphy	PSQI	No	No
Beaudreau et al,^27^ 2008	60	NA	Actigraphy	NA	No	No
Korn et al,^28^ 2009	42	NA	NA	PSQI	No	No
Elliot et al,^29^ 2010	495	NA	NA	Self-report	No	No
Simpson and Carter,^30^ 2010	10	NA	Actigraphy	PSQI	No	No
Fonareva et al,^31^ 2011	20	20	PSG	PSQI	Yes	Yes
Hirano et al,^32^ 2011	31	NA	NA	Self-report	No	No
Kiecolt-Glaser et al,^33^ 2011	58	74	PSQI	PSQI	Yes	Yes
Oken et al,^6^ 2011	31	25	NA	PSQI	No	Yes
Cupidi et al,^34^ 2012	40	150	NA	PSQI	No	Yes
Simpson and Carter,^35^ 2013	15	NA	NA	PSQI	No	No
Merrilees et al,^36^ 2014	22	NA	Actigraphy	PSQI	No	No
von Känel et al,^37^ 2014	126	NA	Actigraphy	PSQI	No	No
Figueiro et al,^38^ 2015	34	NA	Actigraphy	PSQI	No	No
Sakurai et al,^39^ 2015	20	20	Actigraphy	PSQI	Yes	Yes
Simpson and Carter,^40^ 2015	80	NA	PSQI	PSQI	No	No
Sloane et al,^41^ 2015	17	NA	NA	PSQI	No	No
Lathan et al,^42^ 2016	527	NA	Self-report	NA	No	No
Leggett et al,^43^ 2016	158	NA	Self-report	NA	No	No
Wang et al,^44^ 2016	72	NA	NA	PSQI	No	No
Gibson et al,^45^ 2017	15	NA	Actigraphy	PSQI	No	No
Liu et al,^46^ 2018	492	NA	NA	PSQI	No	No

Abbreviations: MDSD, Morin Daily Sleep Diary; NA, not applicable; PSDQ, Pittsburgh Sleep Disorders Questionnaire; PSG, polysomnography; PSQI, Pittsburgh Sleep Quality Index; SPQ, Sleep Problems Questionnaire.

### Meta-analysis

We first computed Cohen *d*, which is a measure of standardized mean difference between caregivers and control groups. Cohen *d* is preferred over other effect size measures (eg, point biserial correlation coefficient) because it is insensitive to differences in group sizes.^47^ We then converted Cohen *d* values to Hedges *g* values (unbiased estimates of effect sizes)^48^ for analysis using a software program (Comprehensive Meta-Analysis, version 3; Biostat).

In the present analyses, study participants were from different countries or regions with various demographic distributions. Although these studies adopted different sampling strategies (eg, recruiting only female caregivers or only spousal caregivers), meta-analyses can handle such variability in sampling, region, and demographic distributions by using a random-effects model, which assumes that the true effect sizes differ across studies based on heterogeneity in study design (compare with fixed-effects model).^48^

Following standard meta-analytic approaches, we assigned weights to individual studies by the inverse of their variances, which is based on the notion that studies with larger variances yield less precise estimates. The final calculated effect size was the estimated average of the true effect sizes. We used the *Q* statistic to assess heterogeneity of effect sizes. The *Q* statistic is the sum of squared deviations of each individual study’s effect size from the overall effect size, weighting individual studies by the inverse of their variances. For models that showed significant heterogeneity, we conducted moderation analyses of the following 4 variables: NHLBI-defined study quality assessment score, mean age of caregivers,^49^ percentage of female participants,^50^ and percentage of spousal caregiver participants.^51^ Moreover, we used the trim-and-fill method to assess and adjust for publication bias.^48^ The trim-and-fill test is a nonparametric method for estimating the number of missing studies that may exist (ie, due to publication bias), with an adjusted effect size that takes those missing studies into account.

### Pooled Analysis

We used pooled analysis to compare studies that included noncaregiver control groups relative to those that only examined caregiver adults. The idea here is that studies that were specifically designed to contrast sleep between caregivers and noncaregivers might elicit different outcomes relative to studies in which measuring sleep was not the primary goal (ie, studies without control groups). Furthermore, we conducted pooled analyses that compared all caregivers from all studies with all noncaregiver controls, with the goal of maximizing sample size and generalizability.^52^ All tests were 2-sided, and results were considered significant at *P* ≤ .05.

## Results

We analyzed 35 studies to quantify the differences in total sleep time and sleep quality between 3268 caregivers (pooled mean age [SD of sample means], 63.48 [5.99] years; 76.7% female) and 696 noncaregiving controls. We also assessed intervention-associated changes in caregivers’ sleep quality.

### Total Sleep Time

Total sleep time estimates in caregivers were similar for actigraphy measures (n = 342; mean [SD], 6.76 [1.18] hours) and self-report measures (n = 1044; mean [SD], 6.68 [1.20] hours) (*t*_1384_ = 1.07; *P* = .28; *d* = 0.07). Therefore, we collapsed across those measures in subsequent analyses. By contrast, PSG estimates of total sleep time (n = 133; mean [SD], 6.18 [1.32] hours) were significantly shorter than actigraphy or self-report estimates (*t*_1517_ = 4.73; *P* < .001; *d* = 0.43), which might reflect that individuals typically sleep poorer than normal during their first night of PSG (“first night effect”^53^). Note that we refrained from meta-analyzing sleep stage data because only 2 studies reported sufficient information to calculate effect sizes on those PSG measures.

**Figure 2.**
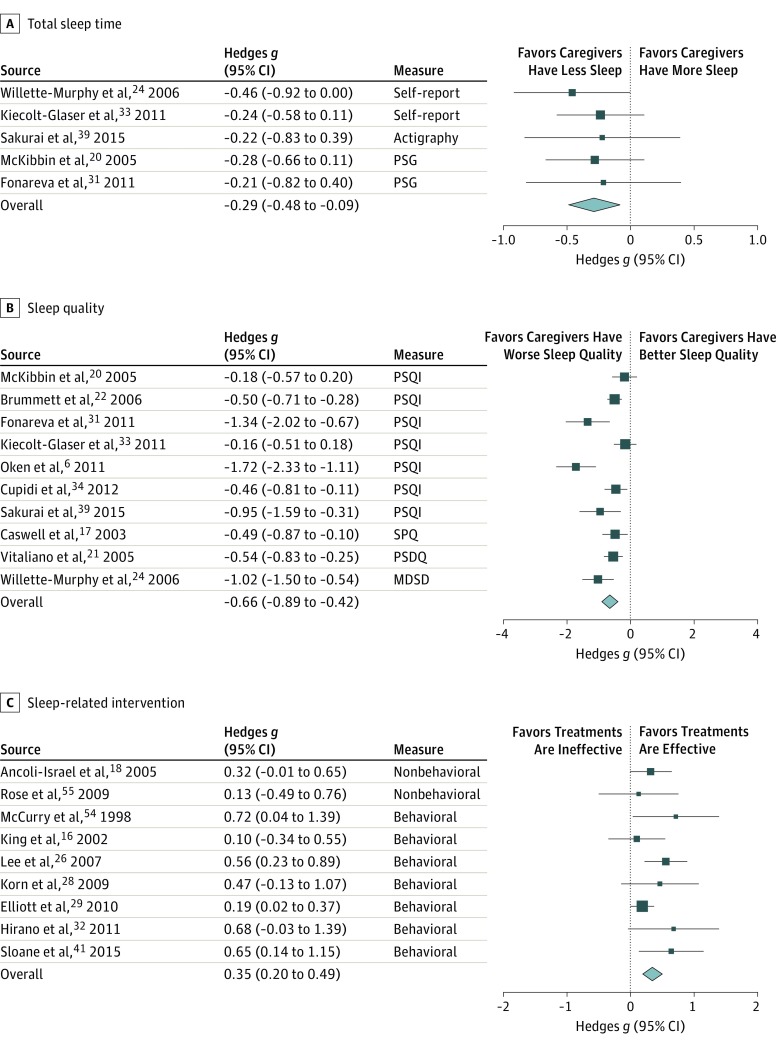
Forest Plots for Total Sleep Time,^20,24,31,33,39^ Sleep Quality,^6,17,20,21,22,24,31,33,34,39^ and Sleep-Related Intervention^16,18,26,28,29,32,41,54,55^ Outcomes A-C, Shown are the mean difference in total sleep time between caregiver (n = 208) and control (n = 191) participants (A), the mean difference in sleep quality between caregiver (n = 594) and control (n = 696) participants (B), and the mean difference in sleep quality between the intervention (n = 469) and control (n = 434) conditions (C). Each square and the line crossing the square represent the standardized effect size and its 95% CI. The size of each square represents the weight of the study. The diamonds represent the mean standardized effect sizes (with their 95% CIs). MDSD indicates Morin Daily Sleep Diary; PSG, polysomnography; PSQI, Pittsburgh Sleep Quality Index; and SPQ, Sleep Problems Questionnaire.

In the studies that included age-matched control groups, Figure 2A shows that caregivers had an average of 0.29 SDs less total sleep time than noncaregiver controls (*g* = −0.29, SE = 0.10; 95% CI, −0.48 to −0.09; *P* = .01). The effect size estimates were similar for PSG and actigraphy/self-report studies (*Q*_1_ = 0.04; *P* = .85). This 0.29-SD effect size translates to approximately 2.42 fewer hours of sleep each week in caregivers, and similar estimates were seen for total sleep time loss in caregivers when calculating the raw mean group differences in actigraphy/self-report studies (3.08 hours each week) and PSG studies (3.50 hours each week). The trim-and-fill test did not yield evidence for publication bias; because there was not significant heterogeneity in effect sizes, we did not conduct moderation analyses of total sleep time data (*Q*_4_ = 0.73; *P* = .95).

When combining actigraphy and self-report data from all caregivers across all study designs (Table 1), the caregivers (n = 1386; mean [SD], 6.70 [1.20] hours) averaged significantly less total sleep time than the noncaregiving control adults (n = 111; mean [SD], 7.14 [1.20] hours) (*t*_1495_ = 3.72; *P* < .001; *d* = 0.37). They also had significantly less mean total sleep time than that in a population-based study^56^ of community-dwelling older adults (6.9 hours of sleep per night) (*t*_1385_ = 6.20; *P* < .001; *d* = 0.17) and significantly less than the recommended minimum sleep duration for adults (7 hours per night^57^) (*t*_1385_ = 9.31; *P* < .001; *d* = 0.25). However, study design was associated with estimates of total sleep time (eFigure 1 in the Supplement). In actigraphy/self-report studies with noncaregiver control groups, the caregivers had shorter sleep durations (n = 622; mean [SD], 6.51 [1.25] hours) than when only caregivers were included (n = 764; mean [SD], 6.85 [1.16] hours) (*t*_1384_ = 5.24; *P* < .001; *d* = −0.28). In PSG studies, caregivers had statistically similar sleep durations regardless of whether noncaregiver control groups were enrolled (*t*_131_ = 1.45; *P* = .15; *d* = 0.27) (eFigure 1 in the Supplement).

### Sleep Quality

Subjective sleep quality was also significantly poorer in caregivers relative to age-matched controls (significant unadjusted *g* = −0.66, SE = 0.12; 95% CI, −0.89 to −0.42; *P* < .001). In the studies with noncaregiver control groups, Figure 2B shows that caregivers’ average sleep quality was 0.66 SDs worse than that of noncaregivers. The effect sizes were similar between studies that used the PSQI (*g* = −0.68, SE = 0.17; 95% CI, −1.02 to −0.34; *P* < .001) and studies that used other subjective measures of sleep quality (*g* = −0.64, SE = 0.14; 95% CI, −0.92 to −0.36; *P* < .001) (*Q*_1_ = 0.60; *P* = .44). After adjusting for publication bias (eFigure 2 in the Supplement), caregivers still had significantly worse sleep quality (*g* = −0.57; 95% CI, −0.84 to −0.31; *P* < .05).

When evaluating study design, we identified that caregivers in the studies with age-matched control groups reported significantly worse sleep quality (n = 397; mean [SD], 6.54 [3.82] on the PSQI) than the caregivers in studies without control groups (n = 1289; mean [SD], 5.28 [3.12] on the PSQI) (*t*_1684_ = 6.66; *P* < .001; *d* = 0.38). Again, the pooled analysis that was inclusive of all caregivers across all studies (n = 1686; mean [SD], 5.58 [3.30]) (Table 1) showed significantly worse PSQI global scores relative to the noncaregiving control data (n = 478; mean [SD], 4.79 [3.17]) (*t*_2162_ = 4.66; *P* < .001; *d* = −0.24). An analysis of component scores showed that the most common deficits in sleep quality for caregivers were difficulties with initially falling asleep (*t*_634_ = 5.17; *P* < .001; *d* = 0.43), poorer habitual sleep efficiency (*t*_634_ = 6.63; *P* < .001; *d* = 0.55), and sleep disturbances (*t*_634_ = 6.09; *P* < .001; *d* = 0.51) (eTable 5 in the Supplement lists full details of the component score analysis).

The sleep quality results showed significant heterogeneity in the true effect sizes across studies (*Q*_9_ = 33.52; *P* < .001). Therefore, we entered caregivers’ mean age, sex distribution (percentage of women), relationship with care recipients (percentage of spouses), and NHLBI-defined study quality assessment scores in meta-regression models as moderators. Effect sizes were not significantly moderated by caregivers’ age (β = 0.03, SE = 0.03; 95% CI, −0.02 to 0.08; *z* = 1.18; *P* = .24), the percentage of spousal caregivers (β = 0.01, SE = 0.01; 95% CI, −0.01 to 0.02; *z* = 0.89; *P* = .37), or NHLBI study quality assessment score (β = −0.003, SE = 0.014; 95% CI, −0.030 to 0.024; *z* = −0.21; *P* = .83). However, studies were more likely to show poorer sleep in caregivers than in noncaregiver controls if the study included a higher percentage of female caregivers (β =−0.02, SE = 0.01; 95% CI, −0.03 to −0.00; *z* = −2.24; *P* = .03; unexplained variance: *Q*_8_ = 25.60; *P* = .001).^50^ To put this association into context, a 10% increase in the proportion of female caregivers was associated with a 0.20 change in Hedges *g* effect size toward poorer sleep quality.

### Intervention Associations

We analyzed intervention-related changes in sleep quality rather than total sleep time because only 3 studies reported sufficient data on total sleep time to calculate effect sizes. The meta-analysis on sleep quality showed that sleep problems in caregivers are not immutable. As summarized in Figure 2C and Table 2, caregivers who received a sleep intervention had better postintervention sleep than caregivers in a control condition (eg, wait-list or preintervention) (unadjusted *g* = 0.35, SE = 0.07; 95% CI, 0.20-0.49; *P* < .001). There was not significant heterogeneity in effect sizes (*Q*_8_ = 9.49; *P* = .30), and the effect size remained significant when statistically adjusting for potential publication bias (*g* = 0.28; 95% CI, 0.13-0.44; *P* < .05) (eFigure 3 in the Supplement). Behavioral interventions, such as sleep hygiene education, stimulus control, and light chronotherapy, were associated with better postintervention sleep quality (*g* = 0.40, SE = 0.10; 95% CI, 0.20-0.59; *P* < .001), and pharmacological and neurostimulation interventions showed a similar albeit nonsignificant pattern (*g* = 0.28, SE = 0.15; 95% CI, −0.01 to 0.57; *P* = .06). The comparison of effect sizes across behavioral and pharmacological/neurostimulation intervention studies was not statistically different (*Q*_1_ = 0.08; *P* = .78).

**Table 2.** Thirteen Intervention Studies in Caregivers

Source	No. of Caregivers	Intervention	Mean (SD)				Included in the Meta-analysis of Intervention	Randomization	ITT Analysis
			Total Sleep Time, h		PSQI				
			Intervention	Control	Intervention	Control			
McCurry et al,^54^ 1998	36	Behavioral intervention	6.4 (0.9)	6.1 (1.0)	7.8 (3.3)	10.6 (4.4)	Yes	Yes	No
King et al,^16^ 2002	94	Moderate-intensity exercise	6.69 (1.16)	6.66 (1.12)	7.14 (3.40)	7.51 (3.67)	Yes	Yes	No
Ancoli-Israel et al,^18^ 2005	63	Cholinesterase inhibitors (galantamine hydrobromide/donepezil hydrochloride) for care recipients	NA	NA	−0.97 (2.96)^a^	NA	Yes	Yes	Yes
McCurry et al,^19^ 2005	36	Sleep hygiene, daily walking, light exposure	NA	7.0 (1.0)	NA	8.6 (3.4)	No	Yes	Yes
Lee et al,^26^ 2007	39	Institutional respite care	6.88 (0.85)	6.72 (0.81)	NA	NA	Yes	No	No
Korn et al,^28^ 2009	42	Polarity therapy	NA	NA	−3.1 (3.9)^b^	−1.4 (3.2)^b^	Yes	Yes	Yes
Rose et al,^55^ 2009	38	Cranial electrical stimulation	NA	NA	7.8	8.2	Yes	Yes	No
Elliott et al,^29^ 2010	495	Resources for Enhancing Alzheimer’s Caregiver Health (REACH) II trial	NA	NA	NA	NA	Yes	Yes	No
Simpson and Carter,^30^ 2010	10	Behavioral intervention	6.88 (1.43)	7.18 (1.19)	5.20 (3.91)	7.80 (4.98)	No	No	No
Hirano et al,^32^ 2011	31	Moderate-intensity exercise	NA	NA	NA	NA	Yes	Yes	No
Figueiro et al,^38^ 2015	34	Tailored lighting	5.71 (0.94)	5.89 (1.21)	6.22 (2.93)	6.70 (2.13)	No	No	No
Sloane et al,^41^ 2015	17	Blue-white light therapy	NA	NA	3.7	5.4	Yes	No	No
Gibson et al,^45^ 2017	15	Bright light therapy, exercise, sleep hygiene education	NA	7.38 (0.82)	NA	6.60 (2.80)	No	No	No

Abbreviations: ITT, intent-to-treat; NA, not available or used a different sleep quality measure; PSQI, Pittsburgh Sleep Quality Index.

^a^ Change score from baseline.

^b^ Within-subject intervention studies were only included in the meta-analysis if means and SDs of change scores were available.

### Study Quality Assessments

**Figure 3.**
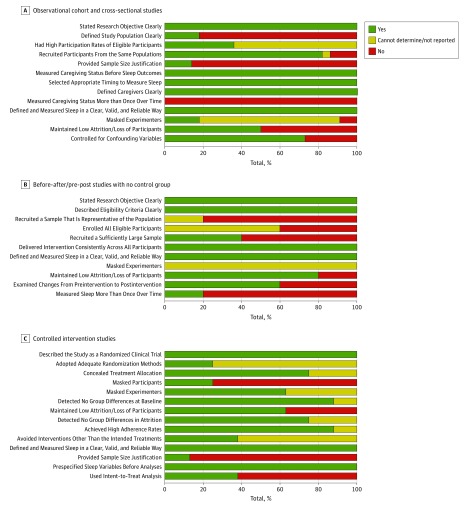
Study Quality Assessment Summary Shown are the strengths and weaknesses of study quality in the sleep and caregiver literature, as defined by National Heart, Lung, and Blood Institute study quality assessments.

In addition to the variability in study design (presence or absence of noncaregiver control group), Figure 3 shows the strengths and weaknesses of study quality in the sleep and caregiver literature, as defined by NHLBI study quality assessments (further details are listed in eTables 2, 3, and 4 in the Supplement). Some strengths included delivering treatments consistently to all participants (100% [5 of 5] of pre-post studies), achieving high adherence rates (87.5% [7 of 8] of controlled intervention studies), and maintaining low attrition rates (80.0% [4 of 5] of pre-post studies and 62.5% [5 of 8] of controlled intervention studies). Despite these positives, only a few studies masked experimenters to caregiving status (18.2% [2 of 11] of observational studies), used adequate randomization methods (25.0% [2 of 8]), or performed intent-to-treat analysis (37.5% [3 of 8]).^13^ Furthermore, there was often limited explicit reporting of sample size justifications, inadequate evidence for sample representativeness, and insufficient description of sample characteristics (eg, length of caregiving and degree of caregiver burden). Collectively, the findings of variability in study design (presence or absence of control groups) and the NHLBI-defined study quality assessments converge to indicate that there is room for greater rigor in planning future studies on sleep in caregivers.

## Discussion

In contrast to the reduced sleep need and caregiver empowerment models, dementia caregivers had poorer perceived sleep quality and shorter sleep durations than age-matched noncaregiver adults and population-based estimates. Caregivers had better sleep quality after undergoing behavioral sleep interventions compared with control conditions, a finding that was consistent with the stress and coping model of caregiving (and inconsistent with externally driven environmental stressor models of caregiving).^11^ We next consider the mechanisms by which caregiving and poor sleep could relate bidirectionally, the theoretical and practical outcomes emanating from caregiver sleep intervention studies, and the future direction of research on caregivers.

### A Bidirectional Account of Sleep and Caregiving

There are several processes by which caregiving for someone with dementia could interrupt sleep. For example, caregivers have heightened stress and increased cognitive burden. They not only have to remember to care for their own needs but also have to remember all of the daily needs of patients with dementia, many of which have clinical implications, such as taking medications and attending medical appointments.^58^ This double to-do list can prolong sleep-onset latency, consistent with the high PSQI subcomponent scores for sleep latency.^19,59^ Furthermore, the night-time awakenings by the patient with dementia could directly contribute to disturbed sleep in caregivers. Such accounts are consistent with the high PSQI subcomponent scores that we observed for sleep efficiency and sleep disturbances.

By the bidirectional account of sleep and caregiving, poorer sleep quality in caregivers should also be associated with worsened ability to provide care. For example, if the caregiver’s physical health has worsened as the result of a sleep loss–induced illness,^60^ then it would be more difficult for the caregiver to perform his or her role effectively. In addition, decreased sleep quality is associated with negative mind-sets, depression, and anxiety, which are in turn associated with the manner in which individuals care for patients with dementia.^34^ Furthermore, numerous experimental, cross-sectional, and other studies have found that short and poor-quality sleep are associated with lower cognitive functioning.^49^ These laboratory findings may have real-world consequences, such as keeping a caregiver from noticing new symptoms, forgetting to administer medications, and being unable to multitask and make reasoned judgments.^5,6^ Perhaps equally important is that an accumulating sleep debt across months and years is associated with quicker cortical thinning and lower clearance of β-amyloid^8^ and tau^61,62^ (via glymphatic system mechanisms). Therefore, poor sleep in caregivers may be associated with increased risk for later developing Alzheimer disease or another dementia.^63^

### Sleep Interventions in Caregivers

The external/environmental-focused view of sleep in caregivers predicts that the caregiver role is so stressful and unpredictable that caregivers would be unable to change their routine in a manner that benefits their sleep. By contrast, the Global Council on Brain Health concluded that all adults can change their behaviors to improve their sleep.^64^ The present meta-analysis results were consistent with the viewpoint of the Global Council on Brain Health in that sleep interventions were associated with better sleep quality for caregivers. Therefore, even though caregivers’ environment is stressful, their sleep quality is more closely linked to their responses to environmental stressors, including whether they engage maladaptive coping techniques, such as increasing alcohol consumption, decreasing exercise, and changing nighttime behaviors that alter sleep quality. Future studies will be needed to address whether sleep duration (in addition to sleep quality) can be improved. With only 3 studies in our meta-analysis reporting intervention outcomes on sleep duration, this research question remains inconclusive.

It is important to note that the sleep interventions for caregivers have been diverse and likely have benefitted sleep through multiple mechanisms. Sleep hygiene education might help improve one’s nighttime routine,^54^ light chronotherapy treatments could entrain circadian rhythms,^41^ daytime exercise can lower the subjective sense of burden and fatigue in caregivers,^32^ and stress management may decrease depressive symptoms.^54^ Behavioral interventions showed statistically similar, but numerically larger, effect sizes for caregivers’ sleep quality than pharmacological interventions (eg, donepezil hydrochloride for care recipients) and cranial electrical stimulation.^18,55^

Future interventions should investigate the effectiveness of Z-drugs (eg, zolpidem tartrate) and orexin receptor antagonists (eg, suvorexant) in caregiver adults. However, because sedative-hypnotic medications often include unwanted adverse effects (eg, decreased responsiveness at night to the care recipient’s needs), behavioral interventions are likely to remain the first-line treatment. Future interventions in caregivers should also consider rates and treatability of clinical sleep disorders, such as obstructive sleep apnea. It is unknown whether adherence to positive airway pressure therapies decreases for dementia caregivers, but this question has implications for caregivers’ cardiovascular and brain health.

### Future Research Directions

In future studies, both caregiver and noncaregiver adults should be recruited in a representative manner from the population, experimenters should be masked to caregiver/control status, and participants should be unaware of the study hypotheses. Put simply, the field should focus on sample representativeness, control groups, and protection against biases. Furthermore, future research should target understanding why sleep quality of women is more adversely influenced by caregiving than that of men. A plausible explanation for the sex difference is that female caregivers tend to report higher caregiving burden and distress than male caregivers.^50^ Future research is needed to test this explanation, as well as other potential psychosocial factors (depression, caregiver burden, grief, and sadness) in moderating or mediating the association between caregiving and sleep. Doing so will inform the development of future targeted interventions.

### Limitations

As with all meta-analyses, the present results should be interpreted in the context of the strengths and weaknesses of the studies composing the sleep and caregiver literature (Figure 3). For example, most studies on sleep in caregivers have used self-report questionnaires rather than objective measures of sleep. This design shortcoming is understandable due to the feasibility and retention challenges of having caregivers leave their home environment to complete in-laboratory PSG (criterion standard sleep measurement). On the one hand, because self-reported sleep duration was greater than PSG-measured sleep duration, it is possible that the actual sleep debt in caregivers was even worse than estimated by the present meta-analysis. On the other hand, all 3 PSG studies used only a single night of in-home PSG and thus may have been influenced by the first night effect.^53^ Repeating PSG for multiple nights in caregivers would yield more accurate estimates of sleep duration and quality.

## Conclusions

Despite the challenges to studying sleep in dementia caregivers, the collective literature indicates that caregivers experience shorter and poorer-quality sleep than age-matched noncaregiver adults. However, such sleep disturbances were less severe in caregivers who received noninvasive behavioral interventions, including sleep hygiene education, light chronotherapy, and bedtime relaxation techniques. Given the long-term, potentially cumulative health consequences of poor-quality sleep,^49^ as well as the increasing prevalence of dementia and the corresponding need for caregivers worldwide,^3^ clinicians should consider sleep interventions not only for the patient with dementia but also for the spouse, child, or friend who will be providing care for that individual.

## Notes

Supplement. **eTable 1.** Characteristics of Studies Included in Meta-analysis (Ordered Chronologically) **eTable 2.** Quality Assessment (NHLBI Cohort and Cross-sectional Studies) **eTable 3.** Quality Assessment (NHLBI Before-After Studies With No Control Group) **eTable 4.** Quality Assessment (Controlled Intervention Studies) **eTable 5.** Pittsburgh Sleep Quality Index Component Scores for Caregivers **eFigure 1.** Study Design Was Associated With Estimates of Total Sleep Time **eFigure 2.** Funnel Plot for the Trim-and-Fill Test on Sleep Quality in Caregivers and Controls **eFigure 3.** Funnel Plot for the Trim-and-Fill Test on Sleep Quality in Intervention and Control Conditions **eReferences.**
