# Supplementary material for: Sleep Duration and Sleep Quality in Caregivers of Patients With Dementia: A Systematic Review and Meta-analysis
Source: JAMA Netw Open. 2019 Aug 23;2(8):e199891. doi: 10.1001/jamanetworkopen.2019.9891 (PMC6714015; doi:10.1001/jamanetworkopen.2019.9891)
Supplement: Supplement. — eTable 1. Characteristics of Studies Included in Meta-analysis (Ordered Chronologically) eTable 2. Quality Assessment (NHLBI Cohort and Cross-sectional Studies) eTable 3. Quality Assessment (NHLBI Before-After Studies With No Control Group) eTable 4. Quality Assessment (Controlled Intervention Studies) eTable 5. Pittsburgh Sleep Quality Index Component Scores for Caregivers eFigure 1. Study Design Was Associated With Estimates of Total Sleep Time eFigure 2. Funnel Plot for the Trim-and-Fill Test on Sleep Quality in Caregivers and Controls eFigure 3. Funnel Plot for the Trim-and-Fill Test on Sleep Quality in Intervention and Control Conditions eReferences. [file jamanetwopen-2-e199891-s001.pdf]

Supplementary Online Content

Gao C, Chapagain NY, Scullin MK. Sleep duration and sleep quality in caregivers of patients with dementia: a systematic review and meta-analysis. *JAMA Netw Open*. 2019;2(8):e199891. doi:10.1001/jamanetworkopen.2019.9891

eTable 1. Characteristics of Studies Included in Meta-analysis (Ordered Chronologically)

eTable 2. Quality Assessment (NHLBI Cohort and Cross-sectional Studies)

eTable 3. Quality Assessment (NHLBI Before-After Studies With No Control Group)

eTable 4. Quality Assessment (Controlled Intervention Studies)

eTable 5. Pittsburgh Sleep Quality Index Component Scores for Caregivers

eFigure 1. Study Design Was Associated With Estimates of Total Sleep Time

eFigure 2. Funnel Plot for the Trim-and-Fill Test on Sleep Quality in Caregivers and Controls

eFigure 3. Funnel Plot for the Trim-and-Fill Test on Sleep Quality in Intervention and Control Conditions

eReferences.

This supplementary material has been provided by the authors to give readers additional information about their work.

© 2019 Gao C et al. *JAMA Network Open*.

eTable 1. Characteristics of Studies Included in Meta-analysis (Ordered Chronologically)

Study	Country	Mean age	%female	%spouse	Study type	Care-recipients' dementia type	Other measures
[a]McCurry, 1998[1]	USA	68.7	78	75	Between-subject intervention	AD, vascular dementia, dementia due to Parkinson's disease, other	Caregiving burden, depression
Wilcox, 1999[2]	USA	62.8	100	53.3	Cross sectional (used baseline data from an intervention study)	AD, multi-infarct dementia, dementia due to Parkinson's disease, anoxia, or Huntington's disease	Caregiving burden, depression, anxiety, stress, anger expression, health
King, 2002[3]	USA	62.72	100	52.94	Between-subject intervention	AD, other	Caregiving burden, depression, stress, exercise adherence, diet, physical activity, cardiovascular reactivity
Caswell, 2003[4]	USA	74.27	52.3	100	Cross sectional	AD, primary degenerative dementia	Caregiving burden, distress, cognition
Ancoli-Israel, 2005[5]	USA	68.56	52.38	73.02	Between-subject intervention	AD	NA
McCurry, 2005[6]	USA	63.28	72.22	58.33	Between-subject intervention	AD	Depression
McKibbin, 2005[7]	USA	72.18	71.23	100	Cross sectional	AD	Functional outcomes of sleep
Vitaliano, 2005[8]	USA	72.2	60	100	Cohort	AD, primary degenerative dementia	Depression, anxiety, hostility, cognition, vital exhaustion, glucose and insulin levels
Brummett, 2006[9]	USA	61	74.3	48	Cross sectional	Not specified	Depression, anxiety, stress, social support
Mausbach, 2006[10]	USA	73.3	65	100	Cross sectional	AD	Depression, medical data
Willette-Murphy, 2006[11]	USA	74.1	100	100	Cross sectional	AD and related disorders	Caregiving burden, mental health, daily activity
Adachi, 2007[12]	Japan	66.2	64.3	54.76	Cross sectional	AD	Caregiving burden
Lee, 2007[13]	UK	67.4	67	79.5	Within-subject intervention	AD, other	Sleepiness, quality of life

© 2019 Gao C et al. *JAMA Network Open*.

Beaudreau, 2008[14]	USA	64.8	100	53.3	Cross sectional	Not specified	Depression, health
Korn, 2009[15]	USA	50	90.48	9.52	Between-subject intervention	Not specified	Depression, stress, worry, quality of life
Rose, 2009[16]	USA	74.23	65.79	100	Between-subject intervention	AD, multi-infarct dementia, other/unknown	Depression, caregiving appraisal
Elliott, 2010[17]	USA	61.29	83.64	42.02	Between-subject intervention	AD	Caregiving burden, depression, frustration
Simpson, 2010[18]	USA	63	NA	50	Within-subject intervention	Not specified	Depression
Fonareva, 2011[19]	USA	64.5	90	70	Cross sectional	Not specified	Depression, stress, sleepiness, fatigue, physiological function, inflammation
Hirano, 2011[20]	Japan	73.7	67.74	NA	Between-subject intervention	AD	Caregiving burden, physical activity
Kiecolt-Glaser, 2011[21]	USA	70.1	71	NA	Cross sectional	AD, other progressive dementia	Depression, childhood trauma, childhood adversity, health behaviors, cytokine and telomere length assays
Oken, 2011[22]	USA	64.5	80.6	74.19	Cross sectional	Progressive dementia	Depression, stress, self-efficacy, mindfulness, personality, fatigue, salivary cortisol
Cupidi, 2012[23]	Italy	67.5	57.5	90	Cross sectional	AD	Caregiver burden, depression, quality of life
Simpson, 2013[24]	USA	58.3	66.7	46.7	Cross sectional	Not specified	Caregiving history, socioeconomic status, work status, health
Merrilees, 2014[25]	USA	61.17	63.64	100	Cross sectional	Behavioral variant frontotemporal dementia, semantic dementia	Emotional distress
von Känel, 2014[26]	USA	74.2	70.6	100	Cohort	AD	Affect, stress, health behaviors
Figueiro, 2015[27]	USA	71.8	79.41	NA	Within-subject intervention	AD and related dementia	Depression, light exposure, rest-activity pattern measured by a daysimeter

© 2019 Gao C et al. *JAMA Network Open*.

Sakurai, 2015[28]	Japan	60 (median)	80	35	Cross sectional	Not specified	Stress, lifestyle, health, autonomic nervous system activity (heart rate variability)
Simpson, 2015[29]	USA	63.26	88.75	50	Cross sectional	Not specified	Depression, stress, subjective health
Sloane, 2015[30]	USA	NA	77	35	Within-subject intervention	Not specified	Caregiver burden, depression, sleepiness
Lathan, 2016[31]	USA	60.1	88	38	Cross sectional	AD, other	Cognition, lifestyle
Leggett, 2016[32]	USA	61.59	87.3	38	Cross sectional	Not specified	Depression, cortical awakening response
Wang, 2016[33]	China	58.25	78	20.8	Cross sectional	AD	Depression, stress, self-efficacy
Gibson, 2017[34]	New Zealand	NA	NA	NA	Within-subject intervention	Not specified	Depression, anxiety, cognition, health
Liu, 2018[35]	China	60.02	55.49	61.38	Cross sectional	AD, frontotemporal lobar degeneration, dementia with Lewy bodies	Caregiving burden, depression, anxiety, health

AD = Alzheimer's Disease
NA = Not available
[a]This study listed sleep disturbance as an inclusion criterion, and therefore, it was only included in the meta-analysis on intervention effectiveness (was not included in analyses on sleep quality/duration in caregivers).

© 2019 Gao C et al. *JAMA Network Open*.

eTable 2. Quality Assessment (NHLBI Cohort and Cross-sectional Studies)

Study	1	2	3	4	5	6	7	8	9	10	11	12	13	14
Wilcox & King, 1999[2]	Y	N	Y	Y	N	NA	NA	NA	NA	NA	Y	NA	NA	NA
Caswell et al., 2003[4]	Y	N	CD	N	N	Y	Y	NA	Y	N	Y	NR	NA	Y
McKibbin et al., 2005[7]	Y	N	CD	N	N	Y	Y	NA	Y	N	Y	NR	NA	N
Vitaliano et al., 2005[8]	Y	N	CD	Y	N	Y	Y	NA	Y	N	Y	Y	Y	Y
Brummett et al., 2006[9]	Y	N	CD	N	N	Y	Y	NA	Y	N	Y	NR	NA	Y
Mausbach et al., 2006[10]	Y	N	CD	Y	N	NA	NA	NA	NA	NA	Y	NA	NA	NA
Willette-Murphy, Todero, & Yeaworth, 2006[11]	Y	N	CD	Y	N	Y	Y	NA	Y	N	Y	NR	NA	N
Adachi et al., 2007[12]	Y	Y	Y	Y	N	NA	NA	NA	NA	NA	Y	NA	NA	NA
Beaudreau et al., 2008[14]	Y	N	Y	Y	N	NA	NA	NA	NA	NA	Y	NA	NA	NA
Fonareva et al., 2011[19]	Y	N	CD	Y	N	Y	Y	NA	Y	N	Y	Y	NA	Y
Kiecolt-Glaser et al., 2011[21]	Y	Y	CD	Y	N	Y	Y	NA	Y	N	Y	NR	NA	Y
Oken et al., 2011[22]	Y	N	CD	Y	N	Y	Y	NA	Y	N	Y	N	NA	Y
Cupidi et al., 2012[23]	Y	Y	CD	Y	N	Y	Y	NA	Y	N	Y	NR	NA	Y
Simpson & Carter, 2013[24]	Y	N	Y	NR	N	NA	NA	NA	NA	NA	Y	NA	NA	NA
Merrilees et al., 2014[25]	Y	N	CD	Y	N	NA	NA	NA	NA	NA	Y	NA	NA	NA
von Känel et al., 2014[26]	Y	N	CD	Y	Y	NA	NA	NA	NA	NA	Y	NA	N	NA
Sakurai et al., 2015[28]	Y	N	CD	Y	N	Y	Y	NA	Y	N	Y	NR	NA	N
Simpson & Carter, 2015[29]	Y	N	CD	Y	Y	NA	NA	NA	NA	NA	Y	NA	NA	NA
Lathan et al., 2016[31]	Y	N	Y	Y	N	Y	Y	NA	Y	N	Y	NR	NA	Y
Leggett et al., 2016[32]	Y	N	Y	Y	N	NA	NA	NA	NA	NA	Y	NA	NA	NA
Wang et al., 2016[33]	Y	N	Y	Y	Y	NA	NA	NA	NA	NA	Y	NA	NA	NA
Liu et al., 2018[35]	Y	Y	Y	Y	N	NA	NA	NA	NA	NA	Y	NA	NA	NA
Percent of Ys	100	18	36	82	14	100	100	NA	100	0	100	18	50	73

CD = Cannot determine, N = No, NA = Not applicable, NR = Not reported, Y = Yes.

© 2019 Gao C et al. *JAMA Network Open*.

eTable 3. Quality Assessment (NHLBI Before-After Studies With No Control Group)

**Study**	**1**	**2**	**3**	**4**	**5**	**6**	**7**	**8**	**9**	**10**	**11**	**12**
Lee et al., 2007[13]	Y	Y	N	CD	Y	Y	Y	NR	Y	Y	Y	NA
Simpson & Carter, 2010[18]	Y	Y	N	CD	N	Y	Y	NR	Y	Y	N	NA
Figueiro et al., 2015[27]	Y	Y	CD	N	Y	Y	Y	NR	Y	N	N	NA
Sloane et al., 2015[30]	Y	Y	N	N	N	Y	Y	NR	Y	Y	N	NA
Gibson et al., 2017[34]	Y	Y	N	CD	N	Y	Y	NR	N	N	N	NA
Percent of Ys	100	100	0	0	40	100	100	0	80	60	20	NA

CD = Cannot determine, N = No, NA = Not applicable, NR = Not reported, Y = Yes.

© 2019 Gao C et al. *JAMA Network Open*.

eTable 4. Quality Assessment (Controlled Intervention Studies)

**Study**	**1**	**2**	**3**	**4**	**5**	**6**	**7**	**8**	**9**	**10**	**11**	**12**	**13**	**14**
McCurry et al., 1998[1]	Y	CD	Y	N	Y	Y	Y	Y	Y	Y	Y	N	Y	N
King et al., 2002[3]	Y	Y	Y	N	NR	Y	Y	Y	Y	Y	Y	N	Y	N
Ancoli-Israel et al., 2005[5]	Y	CD	CD	Y	Y	Y	N	Y	Y	Y	Y	N	Y	Y
McCurry et al., 2005[6]	Y	Y	Y	N	Y	Y	N	Y	Y	NR	Y	N	Y	Y
Korn et al., 2009[15]	Y	CD	Y	N	Y	CD	Y	Y	Y	NR	Y	N	Y	Y
Rose et al., 2009[16]	Y	CD	Y	Y	Y	Y	Y	Y	Y	NR	Y	Y	Y	N
Elliott et al., 2010[17]	Y	CD	CD	N	NR	Y	N	CD	CD	NR	Y	N	Y	N
Hirano et al., 2011[20]	Y	CD	Y	N	NR	Y	Y	CD	Y	NR	Y	N	Y	N
Percent of Ys	100	25	75	25	63	88	63	75	88	38	100	13	100	38

CD = Cannot determine, N = No, NR = Not reported, Y = Yes.

© 2019 Gao C et al. *JAMA Network Open*.

eTable 5. Pittsburgh Sleep Quality Index Component Scores for Caregivers

Study	N of caregivers	N of Controls	Subjective sleep quality	Sleep latency	Sleep duration	Habitual sleep efficiency	Sleep disturbances	Use of sleeping medication	Daytime dysfunction
**Caregiver Groups**									
Wilcox & King, 1999[2]	90	NA	1.19(0.72)	1.16(1.06)	0.84(0.86)	1.08(1.13)	1.47(0.54)	0.81(1.18)	1.14(0.68)
King et al., 2002[3]	94	NA	1.16(0.71)	1.15(1.05)	0.83(0.86)	1.05(1.13)	1.45(0.53)	0.81(1.18)	1.14(0.67)
Adachi et al., 2007[12]	42	NA	1.33(0.69)	1.33(1.14)	0.52(0.74)	0.57(0.94)	0.88(0.45)	0.21(0.72)	0.67(0.61)
Lee et al., 2007[13]	39	NA	1.33(0.77)	1.44(1.07)	1.41(1.02)	1.44(1.25)	1.74(0.55)	0.21(0.61)	1.00(0.56)
Kiecolt-Glaser et al., 2011[21]	58	NA	0.90(0.69)	0.79(0.87)	0.81(0.66)	0.60(0.88)	1.05(0.44)	0.53(1.03)	0.90(0.64)
Cupidi et al., 2012[23]	40	NA	0.93(0.83)	1.25(1.06)	0.95(0.85)	0.40(0.67)	1.33(0.47)	0.20(0.72)	0.70(0.91)
Figueiro et al., 2015[27]	34	NA	1.09(0.98)	1.16(0.96)	1.07(0.87)	0.98(1.09)	1.21(0.51)	0.77(0.52)	0.43(0.59)
Gibson et al., 2017[34]	15	NA	1.13(0.74)	1.07(0.70)	0.69(0.63)	1.23(1.09)	1.20(0.41)	0.64(1.01)	0.93(0.59)
**Control Groups**									
Kiecolt-Glaser et al., 2011[21]	NA	74	0.72(0.65)	0.78(0.80)	0.77(0.65)	0.43(0.76)	1.14(0.56)	0.47(0.95)	0.80(0.64)
Cupidi et al., 2012[23]	NA	150	0.93(0.83)	0.73(0.71)	0.69(0.68)	0.40(0.58)	1.04(0.53)	0.34(0.82)	0.77(0.87)
**Pooled Averages**									
Caregivers	412	NA	1.13(0.75)	1.15(1.02)	0.88(0.83)	0.92(1.05)	1.33(0.50)	0.58(0.99)	0.94(0.67)
Controls	NA	224	0.86(0.78)	0.75(0.74)	0.72(0.67)	0.41(0.64)	1.07(0.54)	0.38(0.86)	0.78(0.80)

© 2019 Gao C et al. *JAMA Network Open*.

Comparison between caregivers and controls	NA	NA	*t*(634) = 4.28, *p* < .001, *d* = 0.35	*t*(634) = 5.17, *p* < .001, *d* = 0.43	*t*(634) = 2.48, *p* = .01, *d* = 0.21	*t*(634) = 6.63, *p* < .001, *d* = 0.55	*t*(634) = 6.09, *p* < .001, *d* = 0.51	*t*(634) = 2.55, *p* = .01, *d* = 0.21	*t*(634) = 2.68, *p* = .01, *d* = 0.22

Values reported as Mean (Standard Deviation).
Range for each component is 0 to 3, with lower values indicating better sleep quality.
NA = Not applicable.

© 2019 Gao C et al. *JAMA Network Open*.

eFigure 1. Study Design Was Associated With Estimates of Total Sleep Time

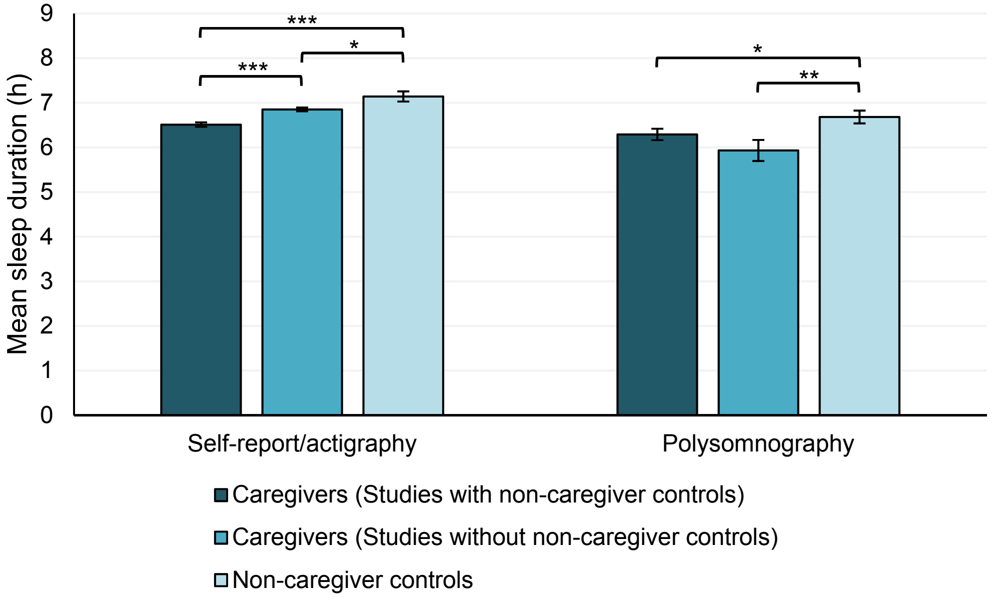

Based on pooled analysis, self-report/actigraphy studies with non-caregiver control groups reported shorter sleep duration in caregivers than studies without non-caregiver controls. The pattern is absent or reversed in polysomnography studies. Error bars: standard error. * $p\leq.05$, ** $p\leq.01$, *** $p\leq.001$.

© 2019 Gao C et al. *JAMA Network Open*.

eFigure 2. Funnel Plot for the Trim-and-Fill Test on Sleep Quality in Caregivers and Controls

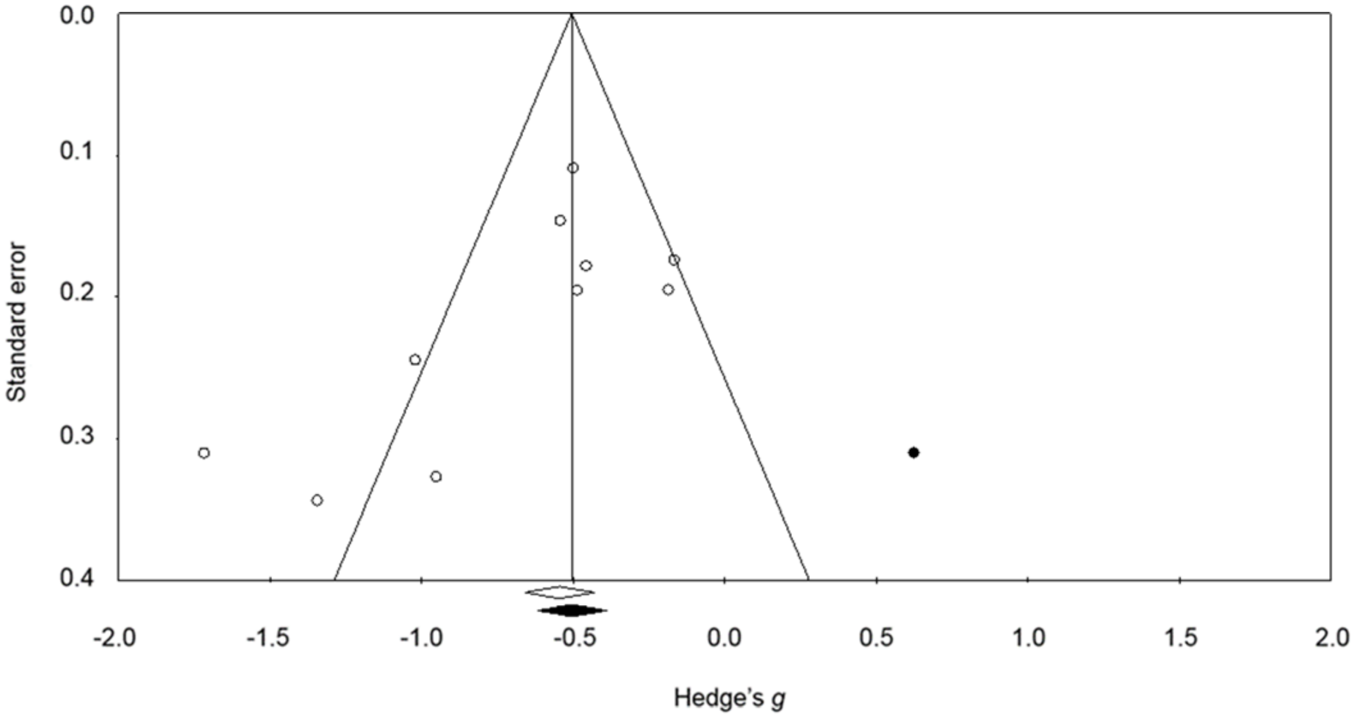

White dots represent observed articles. The white diamond represents observed overall effect size and the 95% confidence interval. Black dots represent missing studies that were added to adjust for publication bias. The black diamond represents adjusted overall effect size and the 95% confidence interval.

© 2019 Gao C et al. *JAMA Network Open*.

eFigure 3. Funnel Plot for the Trim-and-Fill Test on Sleep Quality in Intervention and Control Conditions

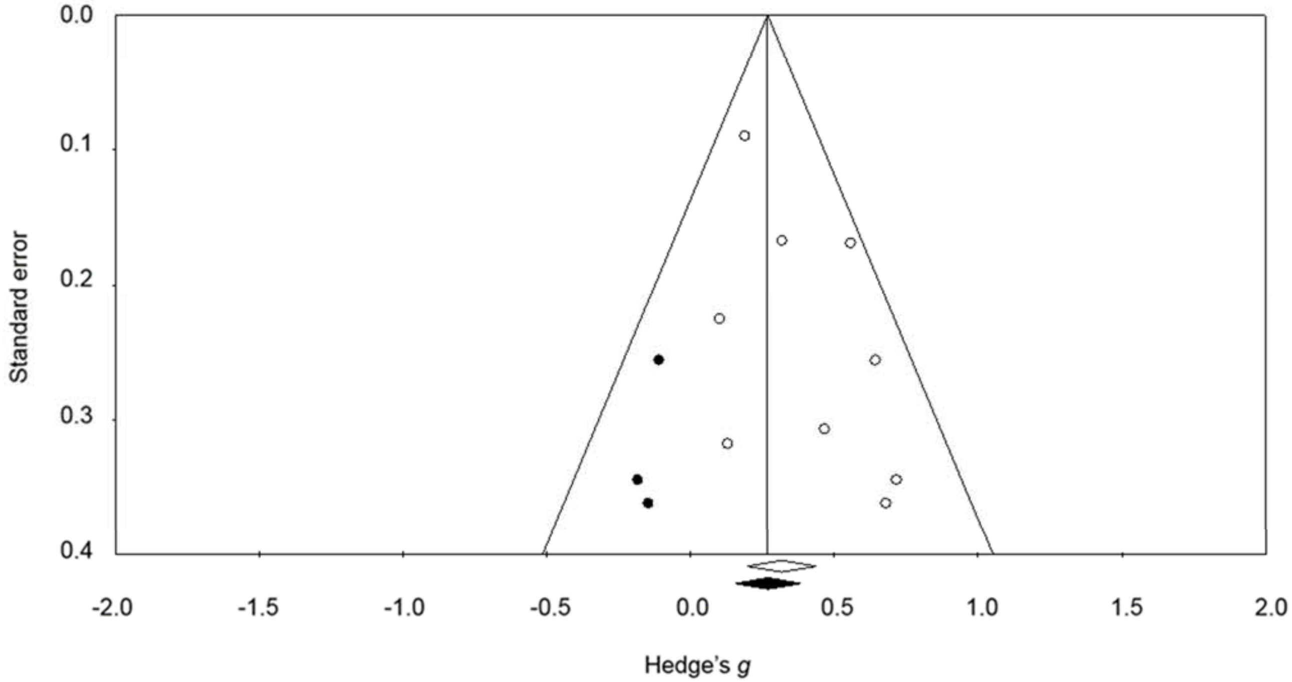

White dots represent observed articles. The white diamond represents observed overall effect size and the 95% confidence interval. Black dots represent missing studies that were added to adjust for publication bias. The black diamond represents adjusted overall effect size and the 95% confidence interval.

© 2019 Gao C et al. *JAMA Network Open*.

eReferences.

1. McCurry SM, Logsdon RG, Vitiello MV, Teri L. Successful behavioral treatment for reported sleep problems in elderly caregivers of dementia patients: a controlled study. *J Gerontol B Psychol Sci Soc Sci*. 1998;53(2):122-129. Medline:9520929 doi:10.1093/geronb/53B.2.P122
2. Wilcox S, King AC. Sleep complaints in older women who are family caregivers. *J Gerontol B Psychol Sci Soc Sci*. 1999;54(3):189-198. Medline:10363041 doi:10.1093/geronb/54B.3.P189
3. King AC, Baumann K, O'Sullivan P, Wilcox S, Castro C. Effects of moderate-intensity exercise on physiological, behavioral, and emotional responses to family caregiving: a randomized controlled trial. *J Gerontol A Biol Sci Med Sci*. 2002;57(1):M26-M36. Medline:11773209 doi:10.1093/gerona/57.1.M26
4. Caswell LW, Vitaliano PP, Croyle KL, Scanlan JM, Zhang J, Daruwala A. Negative associations of chronic stress and cognitive performance in older adult spouse caregivers. *Exp Aging Res*. 2003;29(3):303-318. Medline:12775440 doi:10.1080/03610730303721
5. Ancoli-Israel S, Amatniek J, Ascher S, Sadik K, Ramaswamy K. Effects of galantamine versus donepezil on sleep in patients with mild to moderate Alzheimer disease and their caregivers: a double-blind, head-to-head, randomized pilot study. *Alzheimer Dis Assoc Disord*. 2005;19(4):240-245. Medline:16327351 doi:10.1097/01.wad.0000189052.48688.36
6. McCurry SM, Gibbons LE, Logsdon RG, Vitiello MV, Teri L. Nighttime insomnia treatment and education for Alzheimer's disease: a randomized, controlled trial. *J Am Geriatr Soc*. 2005;53(5):793-802. Medline:15877554 doi:10.1111/j.1532-5415.2005.53252.x
7. McKibbin CL, Ancoli-Israel S, Dimsdale J, et al. Sleep in spousal caregivers of people with Alzheimer's disease. *Sleep*. 2005;28(10):1245-1250. Medline:16295209 doi:10.1093/sleep/28.10.1245
8. Vitaliano PP, Echeverria D, Yi J, Phillips PE, Young H, Siegler IC. Psychophysiological mediators of caregiver stress and differential cognitive decline. *Psychol Aging*. 2005;20(3):402-411. Medline:16248700 doi:10.1037/0882-7974.20.3.402
9. Brummett BH, Babyak MA, Siegler IC, et al. Associations among perceptions of social support, negative affect, and quality of sleep in caregivers and noncaregivers. *Health Psychol*. 2006;25(2):220-225. Medline:16569114 doi:10.1037/0278-6133.25.2.220
10. Mausbach BT, Ancoli-Israel S, von Känel R, et al. Sleep disturbance, norepinephrine, and D-dimer are all related in elderly caregivers of people with Alzheimer disease. *Sleep*. 2006;29(10):1347-1352. Medline:17068989 doi:10.1093/sleep/29.10.1347

© 2019 Gao C et al. *JAMA Network Open*.

11. Willette-Murphy K, Todero C, Yeaworth R. Mental health and sleep of older wife caregivers for spouses with Alzheimer's disease and related disorders. *Issues Ment Health Nurs*. 2006;27(8):837-852. Medline:16938787 doi:10.1080/01612840600840711
12. Adachi H, Ikeda M, Tanabe H, Tachibana N. Determinants of quality of sleep among primary caregivers of patients with Alzheimer's disease in Japan. *Q J Ment Health.* 2007;1(3):61-65.
13. Lee D, Morgan K, Lindesay J. Effect of institutional respite care on the sleep of people with dementia and their primary caregivers. *J Am Geriatr Soc*. 2007;55(2):252-258. Medline:17302663 doi:10.1111/j.1532-5415.2007.01036.x
14. Beaudreau SA, Spira AP, Gray HL, et al. The relationship between objectively measured sleep disturbance and dementia family caregiver distress and burden. *J Geriatr Psychiatry Neurol*. 2008;21(3):159-165. Medline:18503035 doi:10.1177/0891988708316857
15. Korn L, Logsdon RG, Polissar NL, Gomez-Beloz A, Waters T, Rÿser R. A randomized trial of a CAM therapy for stress reduction in American Indian and Alaskan Native family caregivers. *Gerontologist*. 2009;49(3):368-377. Medline:19377083 doi:10.1093/geront/gnp032
16. Rose KM, Taylor AG, Bourguignon C. Effects of cranial electrical stimulation on sleep disturbances, depressive symptoms, and caregiving appraisal in spousal caregivers of persons with Alzheimer's disease. *Appl Nurs Res*. 2009;22(2):119-125. Medline:19427574 doi:10.1016/j.apnr.2007.06.001
17. Elliott AF, Burgio LD, Decoster J. Enhancing caregiver health: findings from the Resources for Enhancing Alzheimer's Caregiver Health II intervention. *J Am Geriatr Soc*. 2010;58(1):30-37. Medline:20122038 doi:10.1111/j.1532-5415.2009.02631.x
18. Simpson C, Carter PA. Pilot study of a brief behavioral sleep intervention for caregivers of individuals with dementia. *Res Gerontol Nurs*. 2010;3(1):19-29. Medline:20128540 doi:10.3928/19404921-20090731-02
19. Fonareva I, Amen AM, Zajdel DP, Ellingson RM, Oken BS. Assessing sleep architecture in dementia caregivers at home using an ambulatory polysomnographic system. *J Geriatr Psychiatry Neurol*. 2011;24(1):50-59. Medline:21320949 doi:10.1177/0891988710397548
20. Hirano A, Suzuki Y, Kuzuya M, Onishi J, Ban N, Umegaki H. Influence of regular exercise on subjective sense of burden and physical symptoms in community-dwelling caregivers of dementia patients: a randomized controlled trial. *Arch Gerontol Geriatr*. 2011;53(2):e158-e163. Medline:20850878 doi:10.1016/j.archger.2010.08.004
21. Kiecolt-Glaser JK, Gouin JP, Weng NP, Malarkey WB, Beversdorf DQ, Glaser R. Childhood adversity heightens the impact of later-life caregiving stress on telomere length and inflammation. *Psychosom Med*. 2011;73(1):16-22. Medline:21148804 doi:10.1097/PSY.0b013e31820573b6

© 2019 Gao C et al. *JAMA Network Open*.

22. Oken BS, Fonareva I, Wahbeh H. Stress-related cognitive dysfunction in dementia caregivers. *J Geriatr Psychiatry Neurol*. 2011;24(4):191-198. Medline:22228825 doi:10.1177/0891988711422524
23. Cupidi C, Realmuto S, Lo Coco G, et al. Sleep quality in caregivers of patients with Alzheimer's disease and Parkinson's disease and its relationship to quality of life. *Int Psychogeriatr*. 2012;24(11):1827-1835. Medline:22652066 doi:10.1017/S1041610212001032
24. Simpson C, Carter P. Dementia caregivers' lived experience of sleep. *Clin Nurse Spec*. 2013;27(6):298-306. Medline:24107753 doi:10.1097/NUR.0b013e3182a87287
25. Merrilees J, Hubbard E, Mastick J, Miller BL, Dowling GA. Sleep in persons with frontotemporal dementia and their family caregivers. *Nurs Res*. 2014;63(2):129-136. Medline:24589648 doi:10.1097/NNR.0000000000000024
26. von Känel R, Mausbach BT, Ancoli-Israel S, et al. Positive affect and sleep in spousal Alzheimer caregivers: a longitudinal study. *Behav Sleep Med*. 2014;12(5):358-372. Medline:24156281 doi:10.1080/15402002.2013.819470
27. Figueiro MG, Hunter CM, Higgins P, et al. Tailored lighting intervention for persons with dementia and caregivers living at home. *Sleep Health*. 2015;1(4):322-330. Medline:27066526 doi:10.1016/j.sleh.2015.09.003
28. Sakurai S, Onishi J, Hirai M. Impaired autonomic nervous system activity during sleep in family caregivers of ambulatory dementia patients in Japan. *Biol Res Nurs*. 2015;17(1):21-28. Medline:25504947 doi:10.1177/1099800414524050
29. Simpson C, Carter P. The impact of living arrangements on dementia caregivers' sleep quality. *Am J Alzheimers Dis Other Demen*. 2015;30(4):352-359. Medline:25425735 doi:10.1177/1533317514559828
30. Sloane PD, Figueiro M, Garg S, et al. Effect of home-based light treatment on persons with dementia and their caregivers. *Light Res Technol*. 2015;47(2):161-176. Medline:26273229 doi:10.1177/1477153513517255
31. Lathan C, Wallace AS, Shewbridge R, Ng N, Morrison G, Resnick HE. Cognitive health assessment and establishment of a virtual cohort of dementia caregivers. *Dement Geriatr Cogn Dis Extra*. 2016;6(1):98-107. Medline:27099613 doi:10.1159/000444390
32. Leggett AN, Liu Y, Klein LC, Zarit SH. Sleep duration and the cortisol awakening response in dementia caregivers utilizing adult day services. *Health Psychol*. 2016;35(5):465-473. Medline:26348500 doi:10.1037/hea0000276
33. Wang PC, Yip PK, Chang Y. Self-efficacy and sleep quality as mediators of perceived stress and memory and behavior problems in the link to dementia caregivers' depression in Taiwan. *Clin Gerontol*. 2016;39(3):222-239. doi:10.1080/07317115.2015.1128503
34. Gibson RH, Gander PH, Dowell AC, Jones LM. Non-pharmacological interventions for managing dementia-related sleep problems within community dwelling pairs: a mixed-method approach. *Dementia (London)*. 2017;16(8):967-984. Medline:26768728 doi:10.1177/1471301215625821

© 2019 Gao C et al. *JAMA Network Open*.

35. Liu S, Liu J, Wang XD, et al. Caregiver burden, sleep quality, depression, and anxiety in dementia caregivers: a comparison of frontotemporal lobar degeneration, dementia with Lewy bodies, and Alzheimer's disease. *Int Psychogeriatr*. 2018;30(8):1131-1138. Medline:29223171 doi:10.1017/S1041610217002630

© 2019 Gao C et al. *JAMA Network Open*.
